# Effects of wilting on silage quality: a meta-analysis

**DOI:** 10.5713/ab.23.0403

**Published:** 2024-02-23

**Authors:** Muhammad Ridla, Hajrian Rizqi Albarki, Sazli Tutur Risyahadi, Sukarman Sukarman

**Affiliations:** 1 Department of Animal Nutrition and Feed Technology, Faculty of Animal Science, IPB University, Bogor 16680, Indonesia; 2 Center for Tropical Animal Studies (CENTRAS), IPB University, Jl. Raya Pajajaran, Bogor 16153, Indonesia; 3 Animal Feed and Nutrition Modelling (AFENUE) Research Group, Department of Animal Nutrition and Feed Technology, Faculty of Animal Science, IPB University, Bogor 16680, Indonesia; 4 National Research and Innovation Agency (BRIN), Jakarta 10340, Indonesia

**Keywords:** Forage, Meta-analysis, Silage, Wilting

## Abstract

**Objective:**

This meta-analysis aimed to evaluate the impact of wilted and unwilted silage on various parameters, such as nutrient content, fermentation quality, bacterial populations, and digestibility.

**Methods:**

Thirty-six studies from Scopus were included in the database and analyzed using a random effects model in OpenMEE software. The studies were grouped into two categories: wilting silage (experiment group) and non-wilting silage (control group). Publication bias was assessed using a fail-safe number.

**Results:**

The results showed that wilting before ensiling significantly increased the levels of dry matter, water-soluble carbohydrates, neutral detergent fiber, and acid detergent fiber, compared to non-wilting silage (p<0.05). However, wilting significantly decreased dry matter losses, lactic acid, acetic acid, butyric acid, and ammonia levels (p<0.05). The pH, crude protein, and ash contents remained unaffected by the wilting process. Additionally, the meta-analysis revealed no significant differences in bacterial populations, including lactic acid bacteria, yeast, and aerobic bacteria, or *in vitro* dry matter digestibility between the two groups (p>0.05).

**Conclusion:**

Wilting before ensiling significantly improved silage quality by increasing dry matter and water-soluble carbohydrates, as well as reducing dry matter losses, butyric acid, and ammonia. Importantly, wilting did not have a significant impact on pH, crude protein, or *in vitro* dry matter digestibility.

## INTRODUCTION

Silage is the fermented and preserved feed made from grass, legumes, or whole crops. Silage quality is influenced by the resident microbial communities, which in turn affect the fermentation process. The type of forage (crop) used, growing conditions, and environmental factors during the wilting period influence the populations of different microbial communities in silos [1]. Wilting significantly impacts silage quality, and its primary objectives, as outlined by Ribas et al [2], include enhancing fermentation quality, mitigating environmental pollution, and minimizing nutrient losses in the form of gases and effluents.

The wilting process affects the moisture content in the silage, thereby influencing the quality of fermentation. Wilting before ensiling is widely practiced in many parts of the world, as it can reduce silo runoff and improve silage fermentation quality. The wilting process can influence both the physical and chemical attributes of the silage [3].

The effect of wilting on silage quality has been examined in various research studies. However, several studies cannot be considered a standard for understanding the impact of wilting on silage quality, as many of them present inconclusive data. For instance, the pH results in the research conducted by Tao et al [4] indicate a decrease after wilting, while the pH results in the study by Kim et al [5] suggest an increase after wilting. Additionally, [6] observed an increase in lactic acid, in contrast to Zheng et al [7], who reported a decrease in lactic acid due to wilting. Furthermore, conflicting results on silage digestibility were reported, with an increase according to Wan et al [8],

Determining whether wilting has a positive or negative effect on silage quality is challenging based on individual research reports. To gauge the overall impact of wilting on silage quality, a generalization process needs to be conducted on existing research using suitable statistical methods. This study aims to assess the impact of wilting on the quality of silage through a meta-analysis method. It is important to note that, while meta-analysis can provide valuable insights, it cannot replace individual research results. The accuracy of individual research results remains robust, aligned with the specific conditions of each study when silage is made, such as the type of forage species utilized, the timing of harvesting, the incorporation of additives, and the adjustment of dry matter (DM) at initial silage making [1,9,10]. These variables play a crucial role in shaping the outcomes of each study and must be considered when evaluating the overall impact of wilting on silage quality.

Meta-analysis is widely recognized as the preferred method for synthesizing research results across disciplines. Its extensive application in numerous fields has illustrated its versatility in consolidating research findings. In a meta-analysis, the data are analyzed, emphasizing the strength or size of an effect rather than the statistical significance of individual studies. The robust results of the meta-analysis can be obtained after incorporating various factors, including sample size, research methodologies, and publication [11,12]. Careful consideration of these factors enhances the reliability and generalizability of the meta-analytical findings, providing a more comprehensive and nuanced understanding of the overall impact being assessed [13,14].

## MATERIALS AND METHODS

### Database development

A database was developed from several types of literature that reported the effect of wilting on silage. The search of the literature was conducted using Scopus with the keywords used being ‘wilting’ and ‘silage/ensiling’. The database was made in August 2023 from the Scopus research database. The selection criteria were: i) English-language articles; ii) direct comparison between wilting and un-wilting silage; iii) comparison of chemical content, bacteria silage population, and DM digestibility and; iv) replication and variance were reported (standard deviation [SD] or standard error or means). These criteria followed the preferred reporting item for systematic reviews and meta-analysis protocol.

All relevant literature titles are collected along with other information. All literature is collected and a database is formed using a data aggregation process. Data aggregation is the arrangement of data from the literature to facilitate the analysis calculations used.

**Figure 1:**
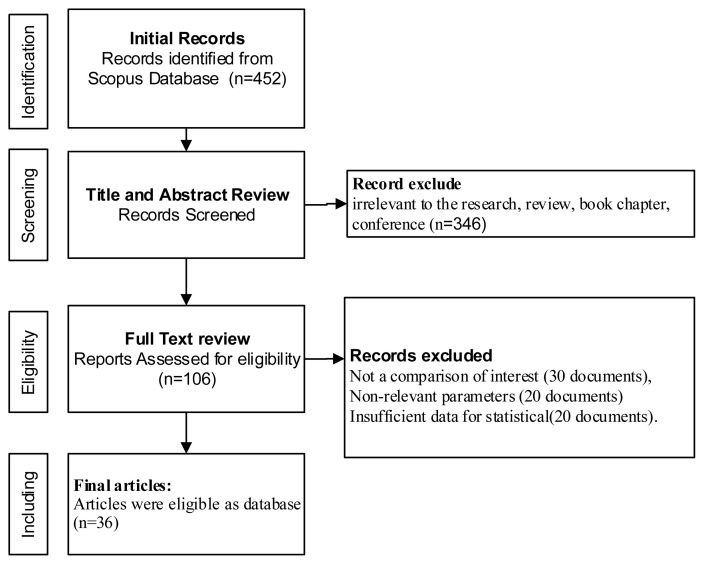
Flow chart of articles selection process based on preferred reporting item for systematic reviews and meta-analysis protocol.

The selection process is shown in Figure 1. The initial search resulted in 452 articles. A total of 346 articles were excluded for several reasons (non-related titles, review articles, or conference proceedings). Hence, the full-text evaluation resulted in 106 articles while 70 articles were excluded due to lack of comparison (n = 30), irrelevant contents or variables (n = 20), and insufficient data (n = 20). The final articles (n = 36) after assessment were considered a database in the meta-analysis shown in Table 1.

### Data extraction

Data were analyzed using the random-effects meta-analysis method as described by Risyahadi et al [15]. The mathematical modeling of one-way random effects follows:


$$y_{i}=\theta +\nu_{i}+{ɛ}_{i}$$

In this equation, *y_i_* represents the effect size (Hedge’s d) for the *i*-th observation, *θ* is the general parameter for the combined effect size, *v_i_* represents the actual variation in the effect size, and *ɛ_i_* is the error for the *i*-th observation.

In brief, the effect size (d) was calculated based on Hedges’ standardized mean difference, with the formula [16]:


$$\text{d}=\frac{\left({\bar{X}}^{E}-{\bar{X}}^{C}\right)}{S}J$$

Where the mean of the experimental or wilting process is ($\bar{X}$^E^), the control group or without wilting process is ($\bar{X}$^C^), and the pooled standard deviation is (S) S defined as:


$$S=\sqrt{\frac{(N^{E}-1)\;{\left(S^{E}\right)}^{2}+(N^{c}-1)\;{\left(S^{c}\right)}^{2}}{N^{E}+N^{c}-2}},$$

and J is the correction factor for the small sample size, i.e.:


$$\text{J}=1-\frac{3}{\left(4\;(N^{E}+N^{c}-2)\right)-1},$$

where: N^E^, sample size of the experimental group; N^c^, sample size of the control group; S^E^, standard deviation of the experimental group; S^C^, standard deviation of the control group. The variance of Hedges’d (V_d_) is described as follows:


$$V_{d}=\frac{\left(N^{E}+N^{c}\right)}{\left(N^{E}N^{c}\right)}+\frac{d^{2}}{\left(2(N^{E}+N^{c})\right)},$$

The cumulative effect size (d_++_) was formulated as follows:


$$d_{++}=\frac{\Sigma_{i=1}^{n}W_{i}d_{1}}{\sum_{i=1}^{n}W_{i}},$$

where: *W_i_*, the inverse of the sampling variance: *W_i_* = 1/vd. The precision of the effect size was described using a 95% confidence interval (CI), i.e. d±(1.96×SD). All the above equations were derived from the study of Sánchez-Meca and Marín-Martínez [17]. Statistical significance was established by verifying that the CI did not encompass a null effect size. Significance was set at a p-value of 0.05. A fail-safe number (Nfs) was calculated to identify publication bias caused by non-significant studies, which were not included in the analysis. Nfs > 5N+10 was considered to provide evidence of a robust meta-analysis model. Nfs was calculated using the method of Rosenthal [18]. The smallest sample size from individual studies was applied as N. Cohen’s benchmarks were used as standard judgment borders for effect size assessment. These benchmarks were: 0.2 for small, 0.5 for medium, and 0.8 for large effect size.

Between-study variance (τ^2^) was estimated using DerSimonian and Laird’s method [19]


$$\tau^{2}=\frac{Q-df}{C}$$

Where Q is the weighted sum square, df is the degrees of freedom, and C is the value. The meta-analysis was conducted using OpenMEE for performance variables. To address potential publication bias from omitted studies, a fail-safe number (Nfs) was computed [18], with Nfs > 5N+10 indicating robustness, applying the smallest sample size from individual studies as N.

## RESULTS

All literature data were incorporated into the data table, and the summarized data were subsequently entered into the data tabulation. Once all the data had been entered, descriptive statistics and various parameters from the database were used to generate Table 2.

Due to conflicting research findings and a small sample size, not all results could be considered reliable owing to publication bias. Briefly, the fail-safe number (Nfs) indicated which studies were suitable to be included in the final robust conclusions. This number expressed how many sample study sizes should be added to change the initial effect size into a negligible variable. If Nfs > 5N+10, where N was the study effect size used to calculate the initial effect size, then the result could be considered as the final robust conclusion [19].

The fail-safe number rules dictate that the robust parameters for assessing silage quality include DM, lactic acid, acetic acid, butyric acid, ammonia, water-soluble carbohydrates (WSC), and acid detergent fiber (ADF). On the other hand, propionic acid, crude protein (CP), pH, and neutral detergent fiber (NDF) are deemed unrobust. Additionally, bacterial populations in silage, such as lactic acid bacteria (LAB), yeast, and aerobic bacteria, as well as *in vitro* dry matter digestibility (IDMD), do not meet the criteria for robust parameters.

This meta-analysis study employed the Q statistics test, τ^2^, and I^2^ to examine heterogeneity. The Q statistic was the weighted sum of the squared values of each study’s effect size deviation from the mean effect size of all studies. The estimate of the population variable tau (τ) was the standard deviation of the overall effect size, and τ^2^ represents the variance of the overall effect size. The I^2^ index was a measure of the proportion of unexplained heterogeneity.

Based on the Heterogeneity Q statistics test, τ^2^, and I^2^, it was observed that some variables exhibited high heterogeneity, while others demonstrated low heterogeneity. Concerning the chemical content of the silage, all parameters displayed excess heterogeneity when Q was higher than the degree of freedom (Nc-1). IDMD and bacterial population of silage also showed high heterogeneity.

Heterogeneity was influenced by several factors, including the number of studies in the meta-analysis, the extent of variation in study effect sizes (between-studies variance), and the amount of variance in the observed effect size for each study (within-study variance). The heterogeneity of this study was high due to different types of forage, additional treatment, and storage time. Furthermore, the differences in wilting time processes influenced the quality of silage, thereby affecting heterogeneity.

The results of the meta-analysis are presented in Table 3, providing a comprehensive evaluation of nutrient content, fermentation quality, bacterial population, and digestibility of silage using Cohen’s methodology. Compared to non-wilted silage, wilting before ensiling significantly increased DM, WSC, NDF, and ADF (p<0.05). Wilting significantly reduced DM losses (p<0.05), as well as lactic acid, acetic acid, butyric acid, and ammonia content. Notably, the pH, CP, and ash content remained unchanged during the wilting process. Furthermore, a meta-analysis showed that the bacterial population of LAB, yeast, and aerobic bacteria, as well as IDMD of silage, were not significantly affected (p>0.05).

## DISCUSSION

### Effects of wilting on dry matter and water-soluble carbohydrates content

Moisture content stands out as a crucial factor influencing silage quality. Excessively wet silage may result in poor fermentation and spoilage, while overly dry silage can lead to inadequate packing and diminished nutritional value. The optimal moisture content for silage falls within the range of 60% to 65% [20].

Dry matter content serves as a more precise indicator of forage moisture levels and is the recommended metric to ensure the correct moisture level for effective silage fermentation and preservation. Digestible energy intake is estimated from DM intake by ruminants, and energy digestibility is obtained from ruminants fed at maintenance levels. Wilting brings about proportional increases in silage DM. Meta-analysis findings indicate that wilting significantly (p<0.05) influences forage silage by elevating DM content and reducing DM loss. This outcome is consistent with numerous experimental studies [21,22]. The increase in DM value results from a lesser decrease in DM losses within the silage. According to Borreani et al [1], the wilting process significantly reduces DM loss, especially in leaves, and is directly associated with the initial DM content of the forage during treatment and the severity of its condition.

The ensiling process is initiated by LAB during fermentation, utilizing water-soluble WSC as energy and carbon sources. Therefore, WSC is crucial for achieving well-preserved silages, with a recommended concentration level of 60 to 70 g/kg of DM [23]. Increasing WSC concentration can enhance fermentation efficiency, promoting faster forage preservation with minimal acid or inoculant use, which can lead to cost savings in silage production.

The meta-analysis reveals that the wilting process significantly increases (p<0.05) WSC content in silage. This finding aligns with the experimental study by Zhang et al [23]. However, it is essential to note that certain researchers have reported no significant impact of wilting on WSC levels [24]. The rise in WSC content can be attributed to the higher concentration of DM. During wilting, the concentration of carbohydrates, including WSC, increases as the forage loses moisture. This elevated concentration results in a higher quantity of WSC relative to the overall DM content of the silage. This finding corresponds with the research by Yahaya et al [25], which indicates that silage with higher DM content contains higher WSC compared to silage with medium and lower DM. Additionally, wilting slows down the respiration of plant cells, leading to reduced carbohydrate consumption. By decreasing respiration, more carbohydrates, including WSC, are preserved in the forage, contributing to higher WSC levels in the silage [1].

### Effects of wilting on pH value and organic acid of silage

The pH value serves as a crucial indicator of silage quality [26]. During the ensiling process, the pH of forage is reduced to a level that inhibits the proliferation of undesirable bacteria, including clostridia, enterobacteria, yeasts, and molds. Conversely, wilting involves elevating the initial DM content of silage to a level that effectively hinders the growth of harmful bacteria, such as clostridia [20].

The meta-analysis results indicated that wilting and non-wilting treatments had no significant impact on pH reduction. This finding aligns with the report by Hao et al [27], who observed that neither wilting nor additive addition to silage affected pH value. The pH value in silage is influenced by microbial heterogeneity, which can alter the lactic acid to acetic acid ratio [28]. Other organic acids, such as propionic acid and butyric acid, also influence pH value. Microbial heterogeneity in silage is further influenced by the type and maturity of the forage, as well as the temperature at ensiling. This variation in silage material and environmental conditions affects microbial populations, which in turn impacts the fermentation process and the resulting pH value [29].

Wilting contributes to the enhancement of silage fermentation quality, particularly by impacting the lactic acid content, which can undergo degradation into other components [30]. This aligns with the findings of the meta-analysis, revealing a decrease in lactic acid levels, accompanied by reductions in acetic-propionic and butyric acids. The wilting process diminishes the overall activity of microorganisms in the silage [31], with a notable decline in clostridium bacteria, as evidenced by a significant reduction in butyric acid levels. This observation is in line with the report by Cole Diepersloot et al [21], which emphasizes that non-wilted forage fermentation yields low butyric acid levels, further emphasizing how wilting can effectively decrease butyric acid content. Low butyric acid levels indicate that silage can be considered high-quality even if lactic acid levels are relatively low and the pH is relatively high, as this suggests effective nutrient preservation [32].

### Effects of wilting on crude protein and ammonia content

While this meta-analysis revealed a slight decrease in CP content in wilted silage compared to non-wilted silage, the difference was not statistically significant (p>0.05). This inconsistency in findings across studies could be attributed to varying conclusions in the research. For example, Kim et al [5] reported lower CP content in wilted silage, while Yahaya et al [25] found higher CP content. Additionally, studies by Hao et al [27] and Wang et al [33] observed both increased and decreased CP levels in treated silages. The potential reduction in CP content due to wilting could be linked to the continued activity of respiration enzymes in surviving plant cells after harvesting, albeit at a reduced level, as evidenced by the nonsignificant decrease in CP observed with wilting.

These findings support the meta-analysis result showing lower ammonia content (p<0.05) in wilted silage compared to unwilted silage, indicating reduced proteolysis activity. The main purpose of wilting before ensiling is to increase DM content, thereby reducing respiration activity and preserving nutrients like CP, which can then be used by animals during feeding. The positive effect of wilting on reducing proteolysis activity has been well-documented by many researchers including [6,23,34,35].

### Effects of wilting on NDF and ADF content

Based on the results of the meta-analysis, wilting in silage leads to a significant increase (p<0.05) in NDF and ADF contents. This finding aligns with the observations made by Reppeto et al [36], who noted a substantial rise in NDF and ADF contents after 8 hours and 10 days of ensiling forage. The significance of this meta-analysis study lies in the variations reported by different researchers. Contrary to these results, Cole Diepersloot et al [21] found no effects on NDF after 30 days of storage, but observed lower levels of NDF for wilting silage after 90 days of storage. Moreover, some studies, such as Zhang et al [23] and Herrmann et al [37], reported decreases in NDF during fermentation, possibly due to solubilization by acid components affecting certain NDF fractions, and this may be linked to the degradation occurring in the wilting process [23]. Despite these differences, it is generally accepted that storage has minimal effects on NDF [38].

### Effects of wilting on silage digestibility

Silage digestibility stands as a critical parameter in determining overall silage quality. The meta-analysis results suggest that wilting treatment does not have a significant impact on IVDMD of silage. This observation aligns with the primary objective of ensiling, which aims to minimize nutrient loss and preserve forage digestibility [39]. The wilting process involves a gradual reduction of moisture, with the goal being not to alter the nutritional content but rather to preserve it as effectively as possible, maintaining the digestible material. This approach differs from rapid drying methods which can lead to diminished nutrient levels and reduced digestibility across various components [40].

### Effects of wilting on silage bacteria

The present meta-analysis indicated no significant influence on microbial populations in silage, including LAB, yeast, and aerobic bacteria (p>0.05). Nonetheless, the expectation for wilting silage was to have a positive effect on bacterial populations, especially in reducing unwanted clostridial populations. This anticipation is due to the diminished oxygen availability resulting from reduced water content in wilted silage, which should inhibit their growth [41]. Different studies have reported varied impacts on the LAB population in wilted silages. For instance, Wan et al [8] observed an increase, whereas Tao et al [4] noted a decrease, and Liu et al [3] recorded both increases and decreases in their respective studies. The unaltered LAB population compared to non-wilted silage might be attributed to the absence of added inoculants. Augmenting the LAB population in silage can be accomplished by including LAB inoculants [33]. According to Kung et al [32], reducing the risk of undesirable bacteria growth can be achieved by ensiling forages at a DM content above 30% to 35%. This is due to the higher DM content, which diminishes the available moisture for bacterial growth and fosters an acidic environment less favorable for harmful bacteria. Additionally, wilting before ensiling further reduces forage moisture, making the environment less conducive to bacterial proliferation [5]. The suppression of bacterial growth due to the wilting process might be indicated by the decrease in lactic acid levels, accompanied by reductions in acetic-propionic and butyric acids, as shown in the current meta-analysis results.

### Regression of dry matter and wilting time

**Figure 2:**
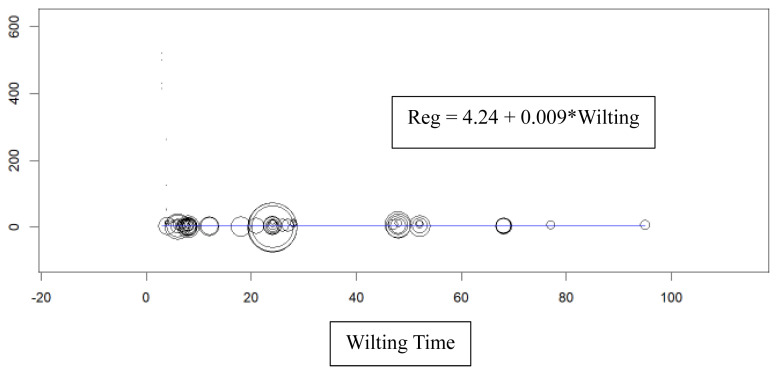
Regression of dry matter and wilting time.

The wilting treatment process positively influences the DM content of forage intended for silage, as demonstrated by the regression equation: DM = 4.24+0.009×Wilting (Figure 2). A more extended wilting duration correlates with a higher DM value. It is crucial to achieve fast wilting with a shorter duration in the field to minimize DM loss [1]. Nevertheless, extended exposure of harvested forage to sunlight may negatively impact the quality of silage as it promotes the growth of undesirable microorganisms. Prolonged wilting durations could also undermine the aerobic stability and nutritional value of silages [42,43].

## CONCLUSION

The present study found that pre-ensiling wilting of forage had a dual impact on chemical composition and silage quality. The process significantly increased DM, WSC, NDF, and ADF, while reducing DM losses, lactic acid, acetic acid, butyric acid, and ammonia. Notably, pH, CP, and ash content remained unchanged during wilting. Additionally, a meta-analysis showed that LAB, yeast, and aerobic bacteria populations, as well as IDMD, were not significantly affected.

## Figures and Tables

**Table 1:** Articles included in the meta-analysis

No.	Reference	Forage type	Additional treatment	Storage time (d)	Wilting time (h)
1	[21]	Bermuda Grass	-	30, 90	4
2	[34]	Oat	With and without inoculant bacteria	112	5
3	[44]	Elephant Grass	With and without Citrus pulp	60	6
4	[4]	Alfalfa	-	45	5.2, 8.5
5	[45]	Grass	-	110	6
6	[35]	Grass Herbage	-	210, 540	24, 48
7	[24]	Comfrey (*Symphytum officinale*)	-	202	24
8	[46]	Perennial ryegrass	Shade and not shade, nonadditive, formic acid, formalin	75	2, 68
9	[47]	Ryegrass, Alfalfa	-	16	3, 4, 8
10	[48]	Grass	Nonadditive, formic acid, and formalin, other additive	6	24
11	[49]	Perennial ryegrass	Low nitrogen, High nitrogen	120	96
12	[50]	Catch crop: a mixture of sunflower, sorghum, peas, Vicia sp. and *Trifolium alexandrinum*	-	7, 14, 28, 98	72, 96
13	[51]	A mixture of *Lolium perenne* L. (81%), *Poa pratensis* L. (9%), and Annual weeds (5%)	-	28	24
14	[52]	Maize	-	40	24, 72
15	[53]	Oat	-	100	14
16	[54]	Wheat cultivar Bet Hashita flowering stage maturity	-	210, 60	8
17	[55]	*Chrysanthemum coronarium* L early bud maturity and late flower maturity	-	120	4, 26, 47, 77, 95
18	[56]	Wheat Forage	-	322	6, 20
19	[57]	Pure sudangrass	Nonadditive, Molasses, *L. plantarum*, *Molasses*+*L. Plantarum*	60	8
20	[22]	Perennial ryegrass	Inoculant, formic acid	70	28, 52
21	[58]	Elephant grass	Nonadditive, cassava meal 7.5% NM, 15% NM, 22.5% NM	60	8
22	[28]	King grass	-	14, 30	12
23	[59]	Perennial ryegrass	Nonadditive, formic acid-formalin	122	48
24	[3]	*Stylosanthes guianensis* Swartz	Temperature 10°C, 20ºC, 30°C, 40ºC	125	6, 12
25	[6]	Guinea grass	Nonadditive, molasess	14, 28, 56	6, 7, 8
26	[5]	Rye grass	-	30	24, 48, 12, 24
27	[33]	*Moringa oleifera* leaf	Nonadditive, *L. plantarum*	60, 120	12
28	[27]	*Broussonetia papyrifera*	Nonadditive, *Enterococcus* durans, cellulase, formic acid	60	3.5
29	[7]	Alfalfa Sanditi, Alfalfa caribou, Alfalfa WL319HQ, Alfalfa 4030	-	14, 28, 56	2, 4
30	[60]	*Medicago sativa* L. 250, *Medicago sativa* L. 350	-	120	4.5, 2.5
31	[23]	Mulberry	Nonadditive, *L. plantarum*, Commercial *L. plantarum*, Cellulase	30	2, 4
32	[61]	Italian ryegrass, festulolium	Nonadditive, *L. Casei*, *L. Bucheri*	120	4
33	[62]	Shorgum	-	30	12
34	[63]	Ryegrass	Additive *L. plantarum*, formic acid		
35	[64]	Whole crop pea	Acid treatment	103	-
36	[65]	Sainfoin	-	120	5–25

**Table 2:** Descriptive statistics of database

Variables	NC	Mean		Min		Max		SD	
			
		Un-wilting	Wilting	Un-wilting	Wilting	Un-wilting	Wilting	Un-wilting	Wilting
Chemical content									
Dry matter (% as fed)	71	19.33	30.03	9.90	13.4	39.80	56.10	1.37	1.79
Crude protein (% DM)	55	14.65	14.46	7.25	6.75	23.02	22.03	1.55	1.51
Water-soluble carbohydrate (% DM)	51	3.38	5.04	0	0.02	12.20	21.80	0.74	0.75
Neutral detergent fiber (% DM)	34	49.42	50.41	19.20	25.20	70.1	70.29	2.50	2.55
Acid detergent fiber (% DM)	27	30.66	31.67	17.20	19.41	41.50	42.50	1.56	1.59
Ammonia (% N)	71	8.24	6.60	0.25	0.10	35.25	17.90	1.05	0.97
Ash (% DM)	15	9.44	9.37	2.26	2.12	16.55	15.90	0.55	0.39
Dry matter losses (% DM)	30	3.45	2.80	0.15	0	13.00	10.10	0.65	0.57
pH and organic acid									
pH	137	4.59	4.61	2.13	2.40	6.77	7.00	0.87	0.64
Lactic acid (% DM)	127	4.91	4.28	0	0	27.64	24.59	0.69	0.53
Acetic acid (% DM)	122	2.16	1.31	0	0	9.34	7.22	0.22	0.22
Propionic acid (% DM)	45	0.38	0.70	0	0	2.12	5.89	0.07	0.11
Butyric acid (% DM)	90	0.62	0.27	0	0	4.41	2.94	0.11	0.10
Microbial population (Log cfu/g)									
Lactic acid bacteria	15	6.86	7.10	4.58	5.00	8.32	8.56	0.49	0.49
Yeast	14	3.98	3.97	0	0	5.40	6.13	0.25	0.24
Aerobic bacteria	14	4.84	5.09	2.19	2.59	7.90	7.90	0.39	0.42
Silage digestibility									
*In vitro* dry matter digestibility (% DM)	18	52.56	50.56	6.4	6.6	79.50	77.00	4.12	3.88

SD, standard deviation; DM, dry matter.

**Table 3:** Meta-analysis on wilting effects on silage quality

Variables	NC	Estimate	Lower bound	Upper bound	Std. error	p-value	τ^2^	Q	Het. p-value	I^2^
Chemical content										
Dry matter	71	4.48	3.63	5.33	0.43	<0.001	8.76	523.42	<0.001	86.63
Crude protein	55	−0.19	−0.53	0.14	0.17	0.27	0.79	113.58	<0.001	52.46
Water-soluble carbohydrate	51	1.34	0.44	2.23	0.46	0.003	8.46	480.63	<0.001	89.59
Neutral detergent fiber	34	0.43	0.01	0.85	0.21	0.04	0.68	60.79	0.002	45.72
Acid detergent fiber	27	0.93	0.38	1.48	0.28	<0.001	1.17	61.20	<0.001	57.52
Ammonia	71	−1.16	−1.69	−0.63	0.27	<0.001	3.87	418.48	<0.001	83.27
Ash	15	0.87	−0.52	2.27	0.71	0.22	5.34	127.62	<0.001	89.03
Dry matter losses	30	−1.59	−2.61	−0.58	0.52	0.002	5.82	164.96	<0.001	82.42
pH and organic acid										
pH	138	0.08	−0.36	0.53	0.23	0.71	4.78	805.02	<0.001	83.23
Lactic acid	127	−0.83	−1.29	−0.37	0.24	<0.001	4.79	746.46	<0.001	83.25
Acetic acid	122	−2.37	−2.90	−1.85	0.27	<0.001	6.19	687.13	<0.001	82.39
Propionic acid	45	−0.38	−1.06	0.29	0.34	0.26	3.68	206.27	<0.001	78.67
Butyric acid	90	−2.46	−3.14	−1.79	0.34	<0.001	7.10	653.99	<0.001	87.00
Microbial population										
Lactic acid bacteria	15	0.40	−0.26	1.06	0.34	0.23	0.89	29.75	0.008	52.95
Yeast	14	0.12	−1.14	1.38	0.64	0.85	3.97	56.60	<0.001	78.80
Aerobic bacteria	14	0.78	−0.02	1.58	0.41	0.06	1.25	29.18	0.004	58.88
Silage digestibility										
*In vitro* dry matter digestibility	18	−0.06	−0.59	0.48	0.28	0.84	0.52	27.85	0.047	38.95
